# Exploring Patients’ Views Toward Giving Web-Based Feedback and Ratings to General Practitioners in England: A Qualitative Descriptive Study

**DOI:** 10.2196/jmir.5865

**Published:** 2016-08-05

**Authors:** Salma Patel, Rebecca Cain, Kevin Neailey, Lucy Hooberman

**Affiliations:** ^1^ WMG Coventry United Kingdom

**Keywords:** Web-based reviews, physician quality, primary care, Internet, quality patient empowerment, quality transparency, public reporting

## Abstract

**Background:**

Patient feedback websites or doctor rating websites are increasingly being used by patients to give feedback about their health care experiences. There is little known about why patients in England may give Web-based feedback and what may motivate or dissuade them from giving Web-based feedback.

**Objective:**

The aim of this study was to explore patients’ views toward giving Web-based feedback and ratings to general practitioners (GPs), within the context of other feedback methods available in primary care in England, and in particular, paper-based feedback cards.

**Methods:**

A descriptive exploratory qualitative approach using face-to-face semistructured interviews was used in this study. Purposive sampling was used to recruit 18 participants from different age groups in London and Coventry. Interviews were transcribed verbatim and analyzed using applied thematic analysis.

**Results:**

Half of the participants in this study were not aware of the opportunity to leave feedback for GPs, and there was limited awareness about the methods available to leave feedback for a GP. The majority of participants were not convinced that formal patient feedback was needed by GPs or would be used by GPs for improvement, regardless of whether they gave it via a website or on paper. Some participants said or suggested that they may leave feedback on a website rather than on a paper-based feedback card for several reasons: because of the ability and ease of giving it remotely; because it would be shared with the public; and because it would be taken more seriously by GPs. Others, however, suggested that they would not use a website to leave feedback for the opposite reasons: because of accessibility issues; privacy and security concerns; and because they felt feedback left on a website may be ignored.

**Conclusions:**

Patient feedback and rating websites as they currently are will not replace other mechanisms for patients in England to leave feedback for a GP. Rather, they may motivate a small number of patients who have more altruistic motives or wish to place collective pressure on a GP to give Web-based feedback. If the National Health Service or GP practices want more patients to leave Web-based feedback, we suggest they first make patients aware that they can leave anonymous feedback securely on a website for a GP. They can then convince them that their feedback is needed and wanted by GPs for improvement, and that the reviews they leave on the website will be of benefit to other patients to decide which GP to see or which GP practice to join.

## Introduction

In England, patients and carers can leave feedback about their experience of a consultation with a general practitioner (GP) using a multitude of methods [1]. These include in-house surveys, the National General Practice Patient Survey, suggestion boxes, surveys for revalidation, the Friends and Family Test, Care Quality Commission ratings, and the NHS Choices and other feedback websites. The number of people leaving ratings and reviews on the Web for products and services in other sectors has exponentially increased [2-4]. A similar type of growth can also be seen, although not to the same magnitude, in the number of patients and carers in England leaving feedback on the Web about their health care experience [5-9].

In 2007, the National Health Service (NHS) introduced the NHS Choices website. Part of the intention and part of the site was designed to encourage patients to provide feedback on health care services [10]. Consequently, on this website, for primary care, patients and carers can (1) view feedback and ratings left by other patients and carers and (2) leave feedback, reviews, or ratings of their health care experience under the GP practice’s name [11]. The former is part of the “choice” agenda that aims to give patients the tools to choose which GP practice to join [12-14]. The latter, the NHS in England states, gives patients a “voice” to air their feedback and concerns independently in the public domain, which they argue will not only increase transparency but also bring improvement and help empower patients [8,10,15]. However, there is little evidence to date to suggest that this has happened.

Research into doctor rating and patient feedback websites is increasing (studies in the United Kingdom [6-8,10,14,16-19], the United States [20-22], Germany [23-27], the Netherlands [28], and Australia [29]). There is some evidence, not always consistent, to suggest that there is an association between Web-based ratings and quality of care [5,6,21,30,31]. In England, although there was some evidence to support a moderate association between patient experience about primary care narrated on a website and via conventional patient surveys, the association with clinical quality of primary care was found to be weak [7].

Studies conducted outside England [25,32-35] have explored what type of patients use patient rating and feedback websites. Two studies conducted in England [36,37] explored patients’ awareness and consideration of their future use of doctor rating websites, as well as some of the demographic predictors for people willing to leave feedback on doctor rating websites. However, none of these studies explored patients’ own views toward patient feedback websites, such as whether they perceive any benefits or risks in relation to leaving feedback on a website, or what may motivate or dissuade them to leave feedback on a website [9]. There is also little understanding of how these attitudes and preferences differ from attitudes and preferences toward other feedback methods. Therefore, the aim of this study was to explore patients’ views toward giving Web-based feedback and ratings to GPs in England, within the context of other feedback methods available in primary care, in particular paper-based feedback cards. The intention is to use the findings from this study to create a questionnaire that could be used across England to explore nationwide public views and understanding toward giving feedback on a website about GPs.

## Methods

### Defining the Context of the Study

The nature of patient feedback websites appears to be evolving quickly. Therefore, to ensure that the research questions developed for this study were up-to-date, and the context within which this study was developed could be understood, before starting this study (in April 2015), the following were outlined: the key stakeholders involved with patient feedback websites (see Multimedia Appendix 1), the characteristics of the Web-based patient feedback platforms available in England (see Multimedia Appendix 2), and the different pathways that a patient may take to use patient feedback websites in England (see Multimedia Appendix 3).

### Data Collection

**Figure 1:**
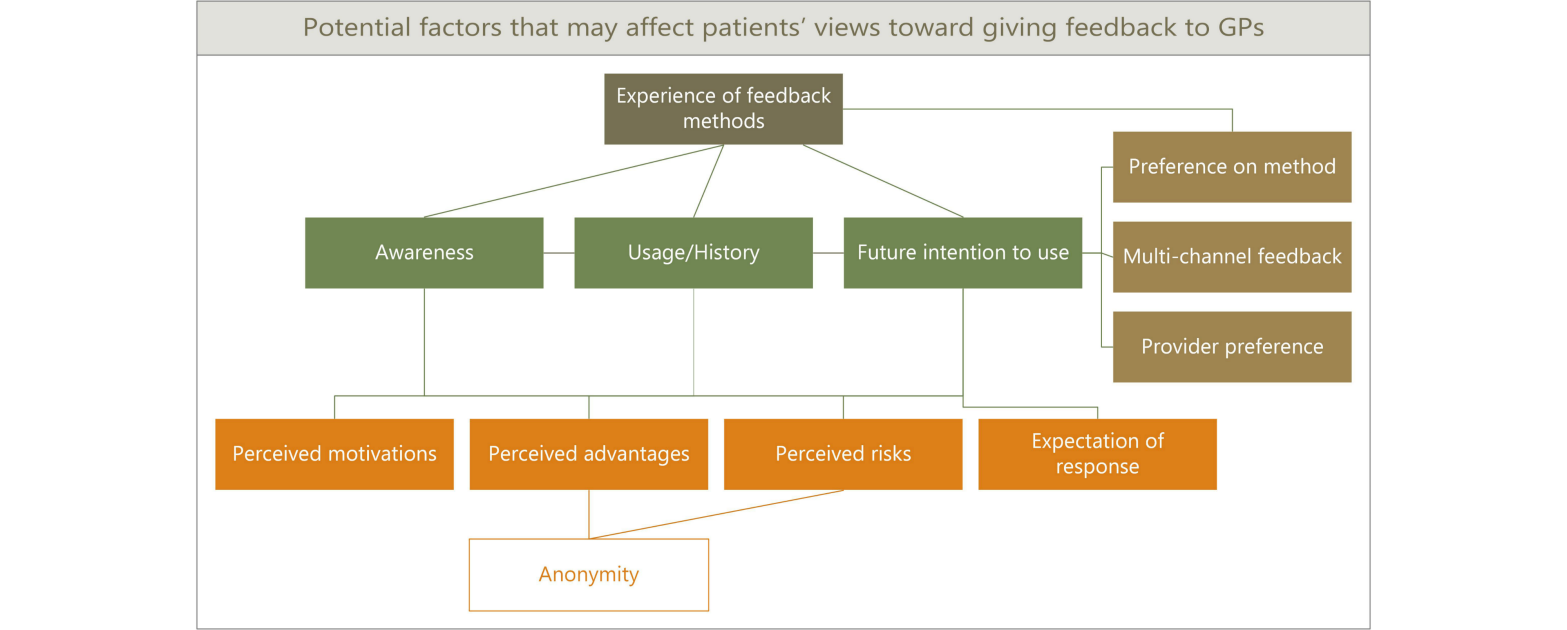
A deductive conceptual framework used to design the topic guide. GP: general practitioner.

This study was exploratory and descriptive in design because there was very little known about patients’ views toward patient feedback websites. Qualitative semistructured interviews were used because this gave the depth required and allowed probing of participants [38]. A deductive conceptual framework was created (see Figure 1) based on existing literature and knowledge gaps, and this, as well as guidance suggested by Bryman [39] and Matthews and Ross [40], was used to design the topic guide (see Multimedia Appendix 4 for a copy of the topic guide). The topic guide was pilot-tested on 2 members of the public before use in the interviews.

Two materials were used in the interviews to provide information to participants. The first was the NHS Friends and Family Test card and the second material contained a screenshot of a GP practice page on the NHS Choices website. Two card sorting exercises were also used to help participants explain which methods they would most prefer to use to leave feedback for GPs. The methods selected were based on feedback methods mentioned by Brown et al [41], Silva [42], and Coulter [43] in patient feedback literature, and are listed in the topic guide (see Multimedia Appendix 4).

### Sampling and Recruitment

Purposive sampling was used to recruit participants so that the sample would represent 3 patients from each age group between the ages of 20 and 80 years. Participants were screened before recruitment to ensure they had at least one consultation with a GP in the past year. A total of 18 participants (10 female; 8 male) were recruited from 4 locations in England: East London, North London, South London, and Coventry. A total of 15 participants were interviewed initially, after which the data were analyzed because the data appeared to have reached close to thematic saturation. Then, 3 further interviews were conducted and analyzed, and the themes that emerged validated and supported the existing themes found. The data were now believed to have reached thematic saturation [44], and therefore no further interviews were conducted.

### Study Interviews

Participants were sent an invitation letter and information sheet beforehand and were interviewed using the topic guide in a private meeting room or at the participant’s home. Informed consent was obtained from all participants. Each interview was on average 30 minutes long and was recorded digitally. The study had ethical approval from the Biomedical and Scientific Research Ethics Committee at the University of Warwick (ref REGO-2015-1472; May 2015).

### Data Preparation and Analysis

The interviews were transcribed verbatim and double-checked for inaccuracies. Transcripts were then transferred to NVivo (QSR International) where they were analyzed using applied thematic analysis [45]. This is a form of inductive (data-driven) thematic analysis that has a pragmatic focus and allows the use of tools appropriate for the analytical process, such as structural coding, quantification, word searches, and deviant case analyses. A structural coding framework consisting of 25 sections was created, which was applied to the first 15 transcripts. Data were then collated relevant to each new code, and a codebook was created in Microsoft Word for each section. As the codebook developed, codes were refined, combined, and deleted from both NVivo and the codebook. Themes were then generated from the codes and reviewed. The 3 new interview transcripts were then added at this stage and went through the aforementioned steps. The themes that emerged supported and validated the existing themes found. The analysis was conducted by the first author (SP), and the codebook was checked for accuracy, internal homogeneity, and external heterogeneity by the second author (RC).

## Results

### Overview

Participants were asked about their views toward giving feedback to GPs, with a focus in particular on patient feedback websites and on paper-based feedback cards. Participants discussed their awareness and past usage of the Web-based and offline modes of feedback to leave feedback for a GP, as well as their attitudes, motivations, and consideration for future use of both websites and paper-based feedback cards. The interviews focused mainly on the NHS Choices website as the Web-based patient feedback mode and the NHS Friends and Family Test feedback card as the offline mode to leave feedback, both of which are available in general practice in England and are generally unsolicited forms of feedback.

This paper presents only the major themes that emerged from the data. The first 4 themes (1-4) were not specific to a method or mode of feedback; rather, they were found in relation to both paper-based feedback and patient feedback websites. The final 3 themes (5-7) were unique to patient feedback websites, and they allude to the additional considerations that patients need to give when considering using websites to leave feedback for a GP.

### Theme 1: Limited Awareness About Methods to Leave Feedback for GPs, Especially on a Website

In this study, 5 participants had given feedback about a GP in the past using non-Web-based methods, and the remaining 13 had not. Interestingly, however, almost half of the participants (n=8) did not know that they could leave feedback about a GP using any method:

I haven’t seen this [NHS Friends and Family Test card] before, probably haven’t looked

P18

Similarly, the majority of participants (n=16) were not aware of the existence of patient feedback websites, and only 1 female participant aged 47 years had experience of giving feedback on a website for a GP. However, more than half of participants (n=12) said they would happily leave feedback about a GP if they were asked to by the GP or the practice, and 13 participants said they may consider giving feedback on a website or on paper in the future.

### Theme 2: Preference for Mode of Feedback Depends on the Nature of Feedback

The majority of participants preferred to give positive feedback directly face-to-face to the GP, and almost half also preferred to give negative feedback directly face-to-face to the GP:

If I was unhappy with my GP I would make an appointment and tell her that I was unhappy. I wouldn’t mess about

P16

**Figure 2:**
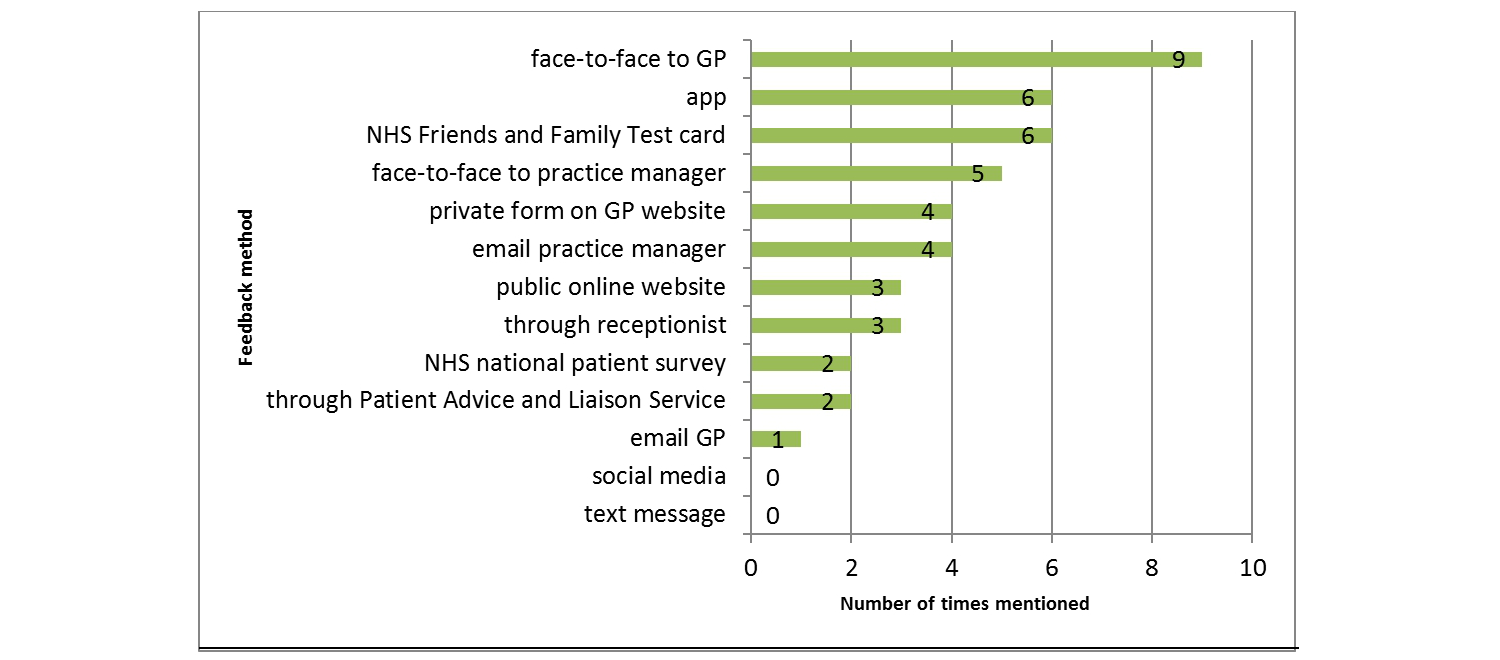
A chart showing the number of times the feedback method was selected (through the card sorting exercise) in participants' 3 most preferred ways to leave feedback for a general practitioner (GP). NHS: National Health Service.

The other methods by which participants most preferred to give feedback were through an app, filling in the NHS Friends and Family Test card, giving the feedback to the practice manager, and leaving the feedback on a private form on the GP website (see Figure 2).

Participants in this study were not keen on using social media (such as Facebook or Twitter) to leave feedback for a GP, emailing or texting the feedback, or using the national patient survey. Among the digital methods the least popular with participants was social media, followed by emailing the GP directly and text messaging. A total of 3 participants (aged between 35 and 55 years) mentioned the website in their top 3 preferred ways to leave feedback about a GP. However, almost all participants added a caveat and said that their preference of which method to use to give feedback to a GP would actually differ depending on the nature of the feedback, that is, whether the feedback was positive or negative:

It depends [on] what feedback you are giving [positive or negative].

P1

### Theme 3: Extreme Experience Is the Primary Driver to Leave Feedback, Both on Paper and on a Website

Almost all of the participants (n=17) described their past experience with a GP as satisfactory or good, and therefore they felt that there was no need for them to leave feedback about a GP:

I suppose it feels a bit silly to make the effort to go to give feedback to say “yeah everything was fine.

P1

However, all participants (n=18) agreed that if they experienced an extreme experience in the future, they would leave feedback for a GP:

If I felt that the level of service [was] exceptionally good or exceptionally poor, I’d be inclined to leave feedback

P15

Furthermore, the majority of participants said they were more likely to leave feedback (on a website or using any other method) when they had experienced an extreme negative experience, rather than an extreme positive experience.

### Theme 4: Patients Need Convincing That Feedback (Both Paper and on a Website) Is Needed and Will Be Used for Improvement

Many participants questioned whether the feedback they leave for a GP would lead to any kind of improvement. Half of the participants (n=9) believed that giving feedback to a GP would not make a difference to the GP’s behavior or practice. A total of 5 participants were unsure whether the GP would even see the patient feedback or respond to it. Furthermore, more than half of participants (n=13) believed that GPs do not want patient feedback, otherwise GPs would ask them to leave feedback for them:

If I was given a card every time I went and they said “can you tick it” then I would tick it and pop it in the box on the way out, but it seems sort of an odd thing to do if I don’t know they [GPs] particularly want it.

P1

A total of 4 participants said that a GP does not need feedback, and a quarter of participants (n=6) said that GPs could utilize their time better by treating patients instead of using their time to read patient feedback. However, more than half of participants (n=12) said they would happily leave feedback about a GP if they were asked to leave feedback by the GP or the practice:

If the paper [to give feedback] was given to me, I would definitely leave feedback.

P7

Furthermore, more than half of participants (n=13) explained that if they were to leave feedback for a GP in the future, their reason for doing so would be to highlight good and bad practice and identify opportunities for improvement:

I think it’s good to highlight good practice, where things go well...but equally with regards to whether [sic] things don’t go as well

P4

### Theme 5: Transparency of Patient Rating and Feedback Websites

A total of 12 participants believed that patient feedback being on a website and in the public domain is advantageous for patients. Among them, 7 participants explained that this was because the public and other organizations could benefit from such feedback because they could evaluate from patient experiences how well GPs and GP practices were performing:

Because it is public isn’t it, and shows the whole world [sic] can see how well the practice is doing.

P6

Other participants (n=4) explained that because the feedback is in the public domain, GPs and the GP practice would take Web-based patient feedback much more seriously than feedback left using other methods, because they would feel more accountable:

With online, because it is in the open, once it is there, it is pretty much like a branding for them, so it’s almost like they have to take it more seriously

P10

Furthermore, 2 participants believed that patient feedback being on a website was advantageous to the GP practice too, because feedback would be easier to collate and there would be less room for error when transferring that feedback to GPs. However, 3 participants believed that these types of feedback websites could be a breeding ground for false complaints, negativity, and abuse:

If you put things that are negative online, it just creates a breeding ground for more...and then becomes a slating of the surgery

P2

One participant said that because of this the GP practice may actually view the feedback left on these websites with skepticism, which, according to her, defeats the purpose of leaving feedback to bring about change or improvement. Similarly, 5 participants (most older than 60 years) questioned the value of patient feedback websites by arguing that these types of feedback websites are not useful to them or to the public:

*What’s the value in people scrolling down and reading I’ve had a particularly good or bad experience? [P9]*

### Theme 6: Concerns About Privacy, Security, and Anonymity of Patient Feedback Websites

More than half of participants (n=10) from all age groups had privacy concerns about leaving feedback on a website and were worried that their identity could be traced, even when leaving feedback anonymously. In contrast, only 2 participants felt that their identity could be revealed if they left feedback for a GP using the NHS Friends and Family Test card. One participant was worried that disclosing her identity when leaving negative feedback on a website or using any other method could risk damaging her relationship with her GP. However, when participants were asked specifically whether they believed leaving negative feedback about a GP would have an impact on their relationship with a GP, most participants believed that GPs were professional, and therefore leaving negative feedback for a GP would not have an impact on their relationship with a GP.

#### Leaving Their Real Name on a Website

A total of 6 participants said they were happy to leave their real name on a website when they left feedback about a GP on a patient feedback website, because they believed their feedback would be more effective with their name on it, because GPs could then use the feedback for improvement:

I always think it is important to [leave one’s name], because if you don’t, then that person can’t get back to you to say how can we improve? Because I always believe it should always be solution focused, so you can’t just sit and moan without thinking how it could be improved, both sides really.

P5

Furthermore, 1 participant mentioned that he would be happy to leave his real name on negative feedback on a website because he could always see another GP in the practice. However, 7 participants from all age groups were not happy to leave their real name on a website because of privacy concerns, because of their need to remain anonymous, and because they were worried that they could be identified by a GP.

#### Leaving Information About Their Diagnoses on a Website

More than half of the participants said that if their diagnosis was a commonly occurring diagnosis they would not mind leaving it on a website. However, if it was quite specific or an embarrassing ailment, they would hesitate to leave it:

I’ve had both my hips replaced, I don’t mind people knowing that...if it was a very personal issue than probably not. Same with online.

P4

#### Naming a General Practitioner When Leaving Feedback on a Website

The majority of participants were happy to name a GP when they left positive feedback about a GP on a website. However, when leaving negative feedback on a website, participants disagreed as to whether a GP should be named. A total of 4 participants said that feedback would be more useful if a GP is named. One of the reasons given was that if the patient wants improvement, the GP needs to be named in the feedback, especially if the GP is part of a larger practice:

There are 18-20 [GPs] working on the same day [in my GP practice], it’s hard to know which doctor you are talking about

P3

However, 7 participants felt that it was unfair to name GPs, because the feedback left on a website could damage the GP’s reputation or personal confidentiality, and it could just be that the GP was having a bad day:

I think they deserve privacy. I live in the public world and I know how that feels, and if I fail I don’t necessarily need it everywhere, and same with them

P6

### Theme 7: Accessibility of Patient Rating and Feedback Websites

Almost half of participants (n=7), all younger than 50 years, believed that a website is more accessible because it is available all the time and can be used from anywhere, and therefore it is also easier to use:

Yeah, cos you can do it [give feedback] any time. You know you don’t have to do it there and then. Or you don’t have to go home and come back to collect something paper-based, you can do it at home, 2am in the morning

P2

Furthermore, 1 participant, who was younger than 30 years, suggested that giving feedback on a website would make it easier for her to be critical of her GP:

I think I would feel more comfortable typing it [i.e., critical feedback] (laugh), it’s just, I don’t know, I think it’s just psychological, I just feel like if I put it down myself [on paper], I wrote it, then it’d be, yeah, I wouldn’t feel as comfortable being as expressive that [sic] I’d like to be. Is that weird?

P3

However, more than half of the participants (n=11) expressed that a website is less accessible. A total of 4 participants (who were all older than 60 years) said this was because they do not have a computer or they do not know how to navigate a website, whereas others who were familiar with the Internet and used the Internet felt they did not want to go on a website for nonwork purposes.

## Discussion

### Principal Findings

In this study, patients as a group are divided about their attitudes toward using feedback and rating websites in the future to leave feedback for a GP. Some patients do not want to leave feedback or do not feel the need to leave feedback in the future (regardless of the method of feedback offered to them), whereas others, who may be willing to leave feedback for a GP, are for or against leaving feedback on a patient feedback website.

The results suggest that some patients may be motivated in the future to leave Web-based feedback rather than paper-based feedback because (1) they can give feedback anytime from anywhere, (2) it allows them to share their experience with the public so others can see what went right or wrong, or (3) they believe that the GP will take Web-based feedback more seriously. On the other hand, however, others suggested that they would not use a website to leave feedback because (1) they cannot use a personal computer or website (mentioned only by participants older than 60 years), (2) they have privacy concerns about leaving feedback on a website, or (3) they believe that feedback left on a website will not be taken seriously by the GP or the practice, because other patients may be abusing the website or using it as a negative breeding ground. These findings can be used by the NHS and patient feedback website providers to effectively target marketing material and address these patient concerns about patient feedback websites that have emerged from this study.

Furthermore, although participants younger than 50 years appeared to perceive giving feedback on a website easier than giving it on paper, this does not mean that they were convinced of the value of giving feedback about a GP on a public feedback website. Privacy and security were important to all of the participants in this study regardless of age, and this suggests that if patients feel a website is not secure enough or will not preserve their anonymity, they will be reluctant to use such a website to leave feedback about GPs. The NHS and other patient feedback website providers need to reassure patients that their websites are secure and will maintain patient privacy.

### Comparison With Prior Work

Since 1978, patient and public involvement has been part of NHS policy, and there has been increasing emphasis on collecting patient experience narratives and feedback both in the NHS and outside it [46]. It was surprising, therefore, that half of the participants in this study were not aware they could leave feedback for or about a GP. In addition, the majority of participants were also not aware of the existence of patient feedback websites. However, the latter is in line with findings from a study by Galizzi et al [36] who found that only 15% of a sample of Londoners were aware of doctor rating websites. This is in contrast to the United States and Germany, where recent studies found that approximately a quarter of respondents had used a physician rating website [25,34]. However, Patel et al [47] suggest this may be partly because of the higher usage of private health care in the United States and Germany.

One of the criticisms of the NHS Choices website in England is that its user-driven content is biased and it contains very few numbers of reviews and ratings, which are not representative of a GP or GP practice’s performance [47]. This was supported by a study in England, which found that less than 1% of all GP consultations had been reviewed on the Web [7], and studies from the United States [30,48-50], Germany [35,51,52], and Australia [29] all indicated that less than 30% of doctors had been rated on the Web. General practitioners in England also suggested that their patients are not aware about the existence of patient feedback websites [47]. The findings from this study appear to support this. However, they also suggest that the lack of awareness and usage among patients is not limited to patient feedback websites; rather, patients appear to have limited awareness of other feedback methods that are present in GP practices too. More positively, however, participants also suggested that this could be reversed if the GP or the practice actively asked them to leave feedback about a GP (rather than by just providing tokenistic methods, such as leaving forms at the reception desk), and this may convince them that their feedback, even if it is mediocre feedback, is of some value to the GP or the GP practice for improvement.

Despite the phenomenal increase in Internet usage and ownership of computers in UK households, there is still a digital divide present in society, where 11% of adults in the United Kingdom in 2015 have never used the Internet [53], and 37% of adults aged 65-74 years and 65% of those older than 75 years do not have access to the Internet at home [54]. This was reflected in our study too, where almost all of the participants older than 60 years said they did not have access to a computer or the knowledge to use such websites. This suggests that some parts of society, mainly the elderly, may be excluded from patient feedback websites, and Trigg [8] proposed that this may be a type of social exclusion for those who most need health care and access to such feedback websites. Interestingly though, even among those who did have access to the Internet in this study and who were familiar with the Internet, a few just did not want to use the Internet for purposes outside of work.

Patients in this study felt that their primary motivation to leave feedback for a GP (irrespective of whether it is on a website or on paper) was to help improve GPs’ professional practice, and this may explain why many in this study preferred to leave feedback directly with the GP or practice, because they believed the GP could then make the necessary changes. This type of motivation is described as “helping the organization” by researchers in the field of consumer behavior, who explore what motivates people to communicate positive and negative sentiments through word-of-mouth about consumer products [55,56]. However, the difference is that this type of motivation was attributed to positive feedback only, whereas in this study, patients attributed it to negative feedback too. This also appears to dismiss the concerns raised in the literature [7,57-59], and by GPs in a previous study [47], that some patients have malicious intentions when they leave feedback on a website.

Two additional perceived patient motivations for leaving feedback on a website were found in this study, and these were exclusive to leaving feedback on a website for GPs. The first of these perceived motivations would fall under the term “altruism” described in the field of e-consumer behavior [60]; this was the ability to benefit other patients and organizations by sharing feedback in the public domain, so that (1) it ensures that others do not share the same negative experience and (2) other patients can use the reviews to decide which GP to see or which GP practice to join. The latter has been part of the “patient choice” agenda in the NHS [11,28], and the NHS argues that this type of “choice” will drive improvement and empower patients [61]. More than half of the participants in this study spoke positively of this advantage; however, there has been considerable criticism of the choice agenda in the literature [12,62].

The second perceived patient motivation to leave Web-based patient feedback mentioned in this study was its collective power to force improvement. This exercising of power over an organization has also been described by Yoo and Gretzel [63] as a motivator for people leaving Web-based travel reviews. Similarly, Ben Bradshaw, a former British Minister for Health, argued that Web-based patient feedback will force doctors to improve their performance and bedside manner out of fear that patients may post on the Web about them [64,65]. However, the majority of GPs in a previous study [47] disagreed that this would bring about a positive change; rather, they believed it would just force GPs to practice more defensively. Davidson et al [66] also found that just because stories about the quality of services appeared in the public domain and affected an organization’s reputation, this did not mean that they would automatically become drivers for improvement in the NHS. Furthermore, 1 participant in this study highlighted that leaving feedback on a website, she believes, will not be taken more seriously by the GP as the feedback may be looked at with skepticism, because patient feedback websites may be seen as negative breeding grounds by GPs. This appears to be supported by some GPs in a previous study [47] who saw little value in Web-based patient feedback and had concerns about them.

Patients’ views about leaving their name on feedback that they would leave on a website in the future were found to be mixed. On the one hand, some patients had concerns about privacy, whereas others suggested the feedback would be more useful to GPs if they as patients left their name on it; 7 GPs in a previous study [47] also believed the same. Similarly, views were mixed about whether GPs should be named in the feedback provided, and a previous study [47] found that GPs preferred to receive practice-based feedback, where they as GPs would not be named by the patient on the feedback left on a website. However, 4 participants in this study believed that feedback would be more useful if the GP is named, because there is no other way to identify the GP, especially if the GP is part of a larger practice. In a study by Patel et al [47], GPs similarly questioned the usefulness of a piece of feedback if it was anonymous to GP and the patient, and remarked that it was difficult to work out who the comment was for and about and therefore could not be used for improvement.

Findings from this study suggest that there is no single most preferred method for patients to give feedback about a GP, and Entwistle et al [67] also found the same in their study with Scottish patients. However, in this study, giving feedback directly to the GP and the practice was the most preferred way for the majority of the participants to leave feedback. This is significant, because it appears to suggest that some patients do not feel the need to formalize the feedback they give about a GP. The results also appear to suggest that if patients feel heard within the practice, they may be less likely to seek out other external ways to leave feedback.

The results from this study also suggest that patients will change their method of giving feedback based on the type of feedback they want to leave (negative or positive) and the type of experience they have. This is significant because it suggests that patient feedback left on a website for a GP—that other patients can then use to make a “choice” of provider—may very well be biased, because it appears to be that patients pick and choose which type of feedback they give on a website and which, for example, they directly tell their GP after a consultation.

All of the participants in this study said that they would consider leaving feedback for a GP (Web-based or using another method) in the future when they had experienced an extreme experience, mainly an extreme negative experience. This appears to support the argument made by GPs in a previous study [47] as well as physician representatives [68,69] that the majority of Web-based patient feedback is extreme negative opinion. This is usually counteracted in the literature with the statement that studies (including [7,10,20,23,30,35,48,59,70-72]) in and out of the United Kingdom have found that the majority of feedback left on physician review websites is positive [73]. The findings from this study appear to contradict that and further suggest that regardless of whether patient feedback is given on a website or not, patients are much more likely to leave feedback when they have experienced an extreme negative experience.

Most participants in this study felt quite comfortable giving negative feedback directly to the GP, and they did not believe leaving negative feedback for a GP would have an impact on their relationship with a GP. This contradicts Dorr and Lipkin’s [74] stance that the doctor-patient relationship is “sacred” and therefore patients would not risk jeopardizing that relationship. However, it appears to support the argument by Kaba and Sooriakumaran [75] that the one-sided power in a doctor-patient relationship is swiftly shifting in the United Kingdom, and the push for patient-centered care means that both parties are now more likely to be involved in decision-making processes.

### Conclusions and Recommendations

The findings of this study appear to suggest that the current low usage of patient feedback websites in England may be partially because many patients do not know that they can leave feedback at all about GPs, Web-based or otherwise, and within the group that does know about leaving feedback for GPs, some do not want to leave feedback, regardless of which method of feedback is offered to them. This is in part because they are not convinced that GPs want patient feedback or need patient feedback. However, the findings also suggest that those patients who do want to leave feedback about a GP would choose the method based on the following: the type of feedback they want to give, whether that particular method of giving feedback was convenient for them, whether they believed the feedback method was secure and appropriate to use, and whether they believed that the feedback would reach the GP using that method and would be used for improvement. These generic factors (found in this study) associated with preference of feedback method may be used by the NHS and other health providers to evaluate whether proposed new methods to collect patient feedback are appropriate and will be effective.

The findings also suggest that patient feedback websites as they currently are will not replace other mechanisms for patients to give feedback to a GP, but they may motivate a small number of patients who have more altruistic motives or wish to place collective pressure on a GP to give feedback on the website. If the NHS or GPs want more patients to leave feedback on the website, the findings suggest they first make patients aware that they can leave anonymous feedback securely on a website for a GP. They could then convince them actively that their feedback is needed and wanted by GPs for improvement and that the reviews they leave on a website will be of benefit to other patients to decide which GP to see or GP practice to join. The findings also suggest that some patients may prefer to give feedback using a Web-based method because it is easier and more accessible, but at the same time they may want their feedback to remain private for the GP or GP practice to view only. Future research will explore this and examine whether the other findings from this study can be found at a population level in England.

### Limitations

Findings from this study provide valuable insight into patients’ views and motivations toward Web-based patient feedback in the context of primary care. However, the findings need to be used with some caution because, even though the data appeared to reach thematic saturation, the sample size for this study was small (n=18), and participants were recruited from 4 locations only. Therefore, it is difficult to conclude to what extent findings can be found in the general population of patients. However, the findings are useful for scoping further research, and future research will examine to what extent findings from this study can be found at a population level in England.

## Appendix

Multimedia Appendix 1 Key stakeholders of Web-based patient feedback for general practitioners in England (as of April 2015).

Multimedia Appendix 2 The different patient feedback websites in England as of April 2015.

Multimedia Appendix 3 Characteristics of websites in England (as of April 2015) where patients can leave feedback for general practitioners publicly.

Multimedia Appendix 4 Topic guide used for the interviews.

## Abbreviations

GP: general practitioner
NHS: National Health Service
