# Supplementary material for: Exploring Patients’ Views Toward Giving Web-Based Feedback and Ratings to General Practitioners in England: A Qualitative Descriptive Study
Source: J Med Internet Res. 2016 Aug 5;18(8):e217. doi: 10.2196/jmir.5865 (PMC4992166; doi:10.2196/jmir.5865)
Supplement: Multimedia Appendix 1 [file jmir_v18i8e217_app1.pdf]

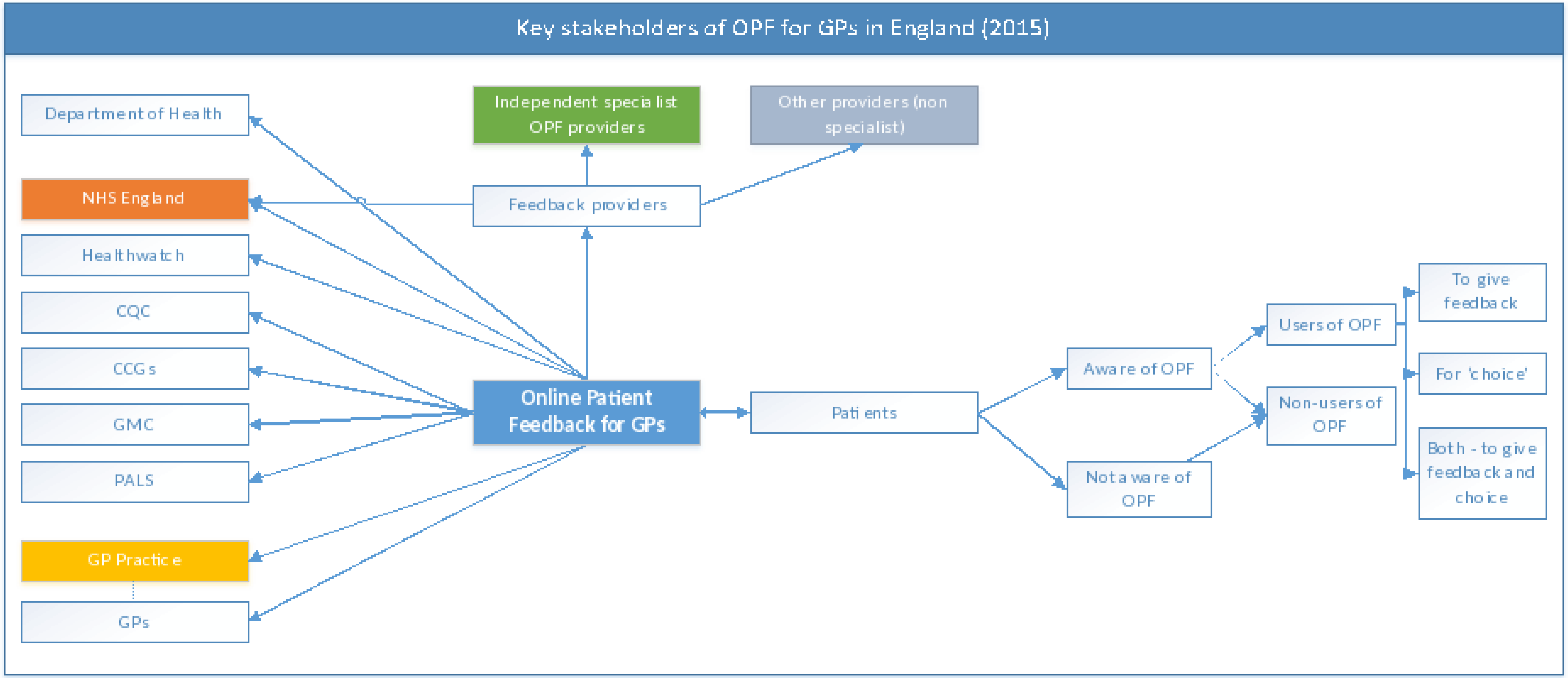

Multimedia Appendix 1: Key stakeholders of online patient feedback (OPF) for GPs in England (as of Apr 2015)
