# Supplementary figures and images for: Exploring Patients’ Views Toward Giving Web-Based Feedback and Ratings to General Practitioners in England: A Qualitative Descriptive Study
Source: J Med Internet Res. 2016 Aug 5;18(8):e217. doi: 10.2196/jmir.5865 (PMC4992166; doi:10.2196/jmir.5865)

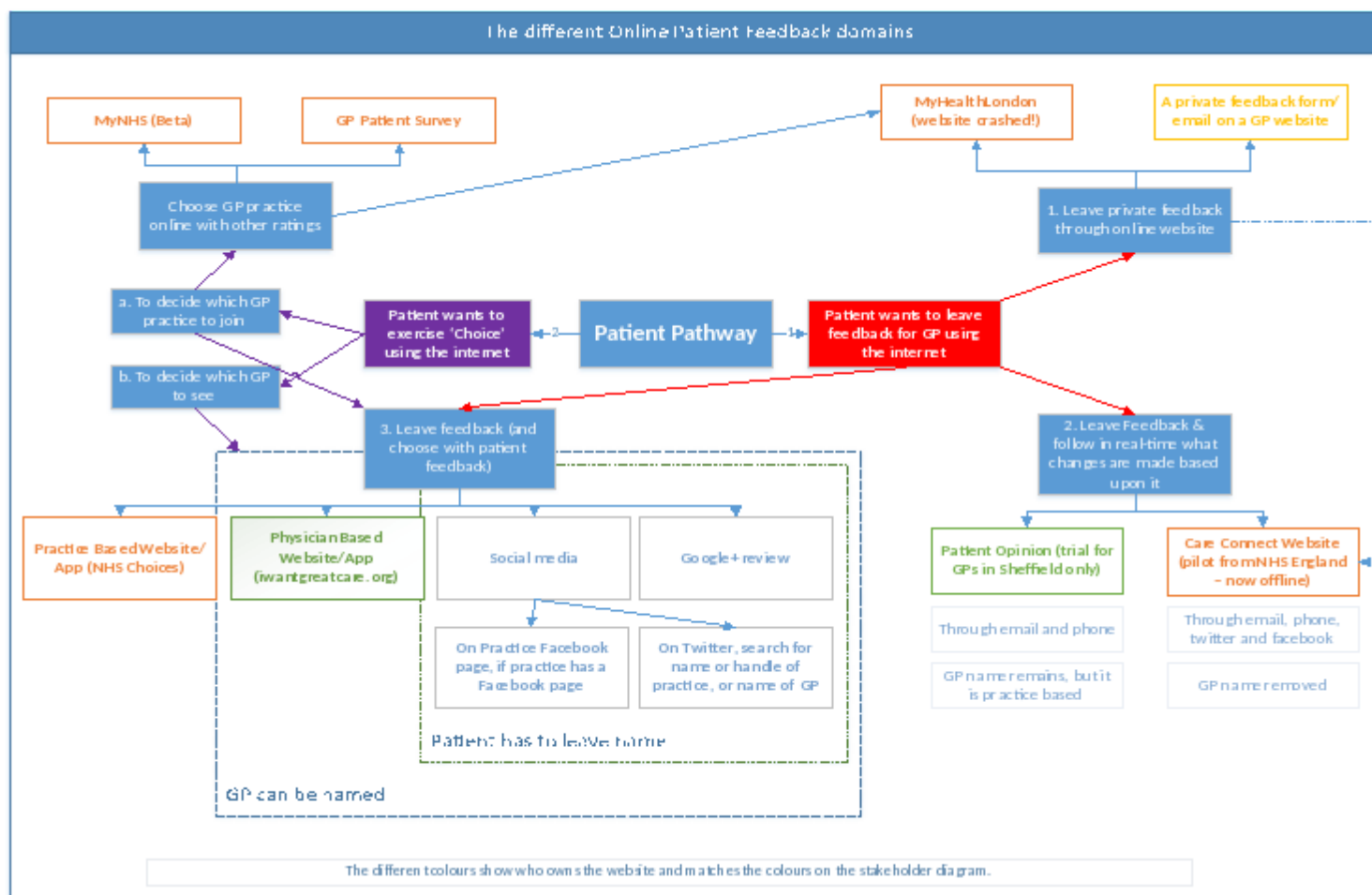

Multimedia Appendix 2 - The different online patient feedback websites in England as of Apr 2015

Supplement: Multimedia Appendix 2 [file jmir_v18i8e217_app2.pdf]
