# Supplementary material for: Exploring Patients’ Views Toward Giving Web-Based Feedback and Ratings to General Practitioners in England: A Qualitative Descriptive Study
Source: J Med Internet Res. 2016 Aug 5;18(8):e217. doi: 10.2196/jmir.5865 (PMC4992166; doi:10.2196/jmir.5865)
Supplement: Multimedia Appendix 3 [file jmir_v18i8e217_app3.pdf]

Multimedia Appendix 3 - Characteristics of online websites in England (as of Apr 2015) where patients can leave feedback for GPs publicly

Name of website	GPs in which region?	NHS Led	Specialist for Health care?	Can patient leave feedback anonymously?	Comments manually moderated before being published	Can a GP be named?	Is the GP alerted of feedback?	Is there an official way for the GP to respond to the feedback?	Can false feedback be challenged and removed?	Tracks real-time change	Multiple channel feedback
NHS Choices	England	[illegible]	[illegible]	[illegible]	[illegible]	[illegible]	[illegible]	[illegible]	[illegible]	[illegible]	[illegible]
iwantgreatcare	UK	[illegible]	[illegible]	[illegible]	[illegible]	[illegible]	[illegible]	[illegible]	[illegible]	[illegible]	[illegible]
Careconnect (now offline)	London	[illegible]	[illegible]	[illegible]	[illegible]	[illegible]	[illegible]	[illegible]	[illegible]	[illegible]	[illegible]
Patient Opinion	Sheffield only	[illegible]	[illegible]	[illegible]	[illegible]	[illegible]	[illegible]	[illegible]	[illegible]	[illegible]	[illegible]
Facebook	World	[illegible]	[illegible]	[illegible]	[illegible]	[illegible]	[illegible]	[illegible]	[illegible]	[illegible]	[illegible]
Twitter	World	[illegible]	[illegible]	[illegible]	[illegible]	[illegible]	[illegible]	[illegible]	[illegible]	[illegible]	[illegible]
Google+	World	[illegible]	[illegible]	[illegible]	[illegible]	[illegible]	[illegible]	[illegible]	[illegible]	[illegible]	[illegible]
