# Supplementary material for: Exploring Patients’ Views Toward Giving Web-Based Feedback and Ratings to General Practitioners in England: A Qualitative Descriptive Study
Source: J Med Internet Res. 2016 Aug 5;18(8):e217. doi: 10.2196/jmir.5865 (PMC4992166; doi:10.2196/jmir.5865)
Supplement: Multimedia Appendix 4 [file jmir_v18i8e217_app4.pdf]

Multimedia Appendix 4

Topic Guide

Interview: Public preference and motivation for giving online patient feedback

1. Introduction

Introduction to research: nature and purpose of research, confidentiality and permission (consent). (Interview length: approximately 45 minutes.)

2. Explain terms used in the interview

- Feedback – relates to review, ratings and complaints that you may give or have given to your GP
- Feedback about your GP – it means in relation to the experience of a consultation that you have had with your GP

- Explain that the questions relate to both positive and negative feedback.

3. GP use

- Roughly, how many times have you had a consultation/appointment with a GP in the past year?

4. Main questions

Awareness of option to give feedback about GP

Are you aware that you can give feedback about your experience of a consultation/appointment with a GP? What methods are you aware of? (If online mentioned, can you name any of the websites?)

Explain the two terms: *Paper-based feedback and online feedback here using vignettes 1 & 2 where appropriate.*

History of 'feedback about GP' – if any

Have you ever given feedback about your GP? (both online and offline)

If no, why not? If yes:

a. How many times roughly have you given feedback about your GP?
b. How did you give the feedback and to whom? (If only one method used)
c. Why did you use this particular method to give feedback?
d. Was the feedback positive or negative?

e. Did you receive a response to the feedback?
f. Were you satisfied with the response?

Paper-based and online feedback

- Would you consider leaving feedback about your GP using paper-based methods? If yes, why? If not, why not?
- If yes, is this the same for both negative and positive feedback?
- Would you consider leaving feedback about your GP online? If yes, is this the same for both negative and positive feedback?

Perceived motives/non-motives for leaving feedback about your GP

- What would be your reasons, if any, for leaving feedback about your GP using paper-based methods? Would these be the same reasons for leaving feedback online?
- What would be your reasons, if any, for not leaving feedback about your GP using paper-based methods? Would these be the same reasons for leaving feedback online?

Perceived motivations for leaving feedback about your GP

- What type of experience, if any, would motivate you to leave paper based feedback about your GP offline? Is this the same for online feedback?
- When are you more likely to leave feedback using paper-based methods: when you've had a negative experience or a positive experience? Is this the same for online feedback?
- Are you more likely to leave paper-based feedback when you've had an extreme experience? Is this the same for online feedback?

Perceived risks of offline feedback, if any

- Do you believe there may be risks to you in leaving feedback online about your GP? If yes, what are they and why? If no, why not? Would the same risks/non-risk apply for leaving feedback through paper-based methods?

Anonymity when leaving feedback

- Would you be happy to leave your real name when you leave feedback about your GP online? If yes, why? If not, why not? Is this same for leaving your name on a paper-based card?

Relationship with GP

- Do you think your relationship with your GP will change if you leave your name on a piece of paper-based feedback you have left for him/her? If yes, how? If no, why not? Is this the same for leaving it online?

Naming GP

- Would you like to name your GP when leaving feedback about him/her online? If yes, why? If no, why not? Is this the same for when you leave feedback through paper-based methods?
- If you had to leave negative feedback online, do you think it is fair to 'name and shame' your GP online? Do you think anything would happen as a result of naming your GP online?
- Do you think it is fair for GPs to ask you not to mention them by their name when you leave feedback about them online? If yes, why? If not, why not?

Content of online feedback for GP

- Imagine you had to leave feedback through a paper-based method. Would you prefer to leave it by (four options):
    1. answering closed ended questions like ticking boxes yes/no
    2. writing sentences about your experience
    3. mixture of both
    4. Or something else? If so, what?

Would this be the same for leaving feedback online?

- Would you consider leaving information about your diagnosis for example in the feedback that you may leave online? *(personal information)* How about on paper?

Expectation of GP response/action to feedback

- Do you think your GP will welcome any feedback you leave for him/her? If yes, why? If no, why not? Would this be the same for both paper-based and online?
- If you left feedback about your GP online, do you expect your GP to do anything with that feedback? What do you expect them to do? Is this the same as when you leave feedback on paper?
- *Do you expect your GP to respond to any feedback you leave using online methods? If yes, why? If no, why not? Is this the same for paper-based methods?*
- Do you think your GP, if needed, will make any changes based on any feedback you leave for him/her using paper-based methods? If yes, why? If no, why not? Is this the same for feedback you leave online?
- If you felt your paper-based feedback required an action by your GP, would you then want to track what exactly happens to your feedback? If yes, why? If no, why not? Is this the same for feedback you leave online too? *(real-time feedback)*

Provider preference

- If you had the option, which of the following three organisations would you prefer to leave paper-based feedback with about your GP: 1) the GP practice 2) an independent organisation 3) a NHS organisation? Why? Is this the same for online too?
- From the three aforementioned methods, which provider would you trust more to do something with your feedback? And why? Is this the same for online and paper-based feedback?

Specific to Online feedback

- Do you believe leaving feedback online will be more effective than leaving it through paper-based methods? If yes, why? If no, why not?

- Do you think your GP will take the feedback you leave online more seriously that the feedback you leave through paper-based methods? If yes, why? If no, why not?

- Do you think you would find it easier to leave feedback online than through other methods? If yes, why? If no, why not?

- Say you had an extreme negative experience when you saw a GP, are you more likely to write to the GP practice with that feedback or are you more likely to leave that feedback online?

- Say you had an extreme positive experience when you saw a GP, are you more likely to write to the GP practice with that feedback or are you more likely to leave that feedback online?

<u>Other</u>

Multi-channel feedback

- Have you ever left feedback for your GP through social media? If yes, how and why? If no, why not?
- If you could leave feedback about your GP on Facebook, would you consider it? If so why? If not, why not?
- Do you want to have the option to be able to leave feedback about your GP through Facebook? If yes, why?
- If you could leave feedback about your GP on Twitter, would you? If so why? If not, why not?
- Would you like to have the option to be able to leave feedback about your GP through Twitter? If yes, why?
- Would you like to have the option to give feedback about your GP through an app on your phone? If yes, why? If no, why not?

- Would you like to have the option to give feedback about your GP by text messaging? If yes, why? If no, why not?
- Would you like to have the option to give feedback about your GP by telephone? If yes, why? If no, why not?

Overall preference on best method to give feedback about GP

- From the following thirteen methods, which three methods would you most prefer to use to leave feedback about your GP (use vignette 3)?

(The options are: 1) face to face directly to your GP 2) paper-based form that you fill in and place in a comments box at the surgery 3) face to face to the practice manager 4) through the receptionist 5) through the PALs 6) email the practice manager 7) public online website 8) text message (to practice) 9) through an app, and 10) through social media 11) Email the GP directly 12) NHS national patient survey 13) private form on GP website

   a. Why?
   b. Which three methods would you least prefer to use to leave feedback about your GP? Why?
   c. Would you want to use a different method to give negative and positive feedback, or is it the same?

- From the following seven methods you could use to give feedback about your GP, could you rank which you prefer the most? (use vignette 4) The options are 1) email GP 2) email practice 3) online website 4) text message 5) through an app, and 6) social media 7) private form on GP website
   a. Why have you ranked them in this way? (most preferred, and least preferred)
   b. Would you want to use a different method to give negative and positive feedback, or is it the same?

Experience of rating websites

- Have you ever used a rating website? (Like trip advisor or amazon reviews?)
- What was your experience of it?
- Have you ever left feedback on a rating website? If yes, why? If no, why not?

Intention to give feedback, online and offline

- In the near future, do you intend to give feedback about your GP?
   a. If yes, would that be online or using paper-based methods? Why?
   b. If no, why not?
- Has this interview changed your knowledge about giving feedback online to your GP?

5. Participant background

Age (note gender down)

6. Ending - Any further things they'd like to add and thank them.

Would you be interested in taking part in further research?

Do you know anyone else that might be interested in taking part in this study?
